# Re-Envisioning Global Health Competencies for the African Region Aligned with Local Health Needs and Resources

**DOI:** 10.5334/aogh.3844

**Published:** 2022-10-20

**Authors:** Ritika Tiwari, René English, Kerrin Begg, Usuf Chikte

**Affiliations:** 1 Division of Health Systems and Public Health, Department of Global Health, Faculty of Medicine and Health Sciences, Stellenbosch University, Francie van Zijl Drive, Tygerberg, Cape Town, PO Box 241, Cape Town, 8000, South Africa

**Keywords:** global health, competencies, Africa, education, public health

## Abstract

**Background:**

While many Global Health programs aim to address health inequalities within and between HICs and low- and middle-income countries (LMICs) there is a need to establish new Global Health academic programs within the growing trend towards ‘internationalization of higher education’.

**Objective:**

This study was undertaken to re-envision Global Health competencies for the African region context with respect to the local health needs and availability of resources.

**Methods:**

This study was undertaken over a period of four years from 2017 till 2020. A three-pronged strategy was undertaken to scan, scope, distil and develop a set of Global Health domains and competencies for the African region. Strategy 1 encompassed an environmental scan of Global Health competencies (2017–2019), and a literature review (2017–2020); strategy 2 comprised a scoping of education programs in Global Health (2018–2019); and strategy 3 involved an interest-group discussion in a face-to-face conference.

**Findings:**

Seven core and four cross-cutting global health competency statements were developed for the African region. The core competency statements included following domains: global health systems and international relations; global evidence ecosystem; role of international organizations; universal health issues; intellectual property rights; responses to issues affecting different at-risk groups; local, national, and international policy and economic context affecting global health. The four cross-cutting competency statements included following domains: digital and academic literacies; quantitative and qualitative research; policy and funding allocation resources; ethical conduct of global health practice and research global health.

**Conclusion:**

There is a need to enable higher education institutions (HEIs) from the Global South to offer global health qualifications with a set of competencies that better approximate solutions to contextualised problems – not only to students from the Global South but also from the Global North. The global health competencies developed in this research study will enable African HEIs to offer global health education in a more pragmatic manner.

## Introduction

In 2022, the World Health Organization (WHO) celebrated the World Health Worker Week 2022 – ‘Build the Health Workforce Back Better’ [1]. The theme emphasized on investing in *competency-based* education for achieving universal health coverage. The term ‘competency’ is sometimes used interchangeably with competence. In literature, at times the term ‘competences’ is used, and at times ‘competency’ or the plural ‘competencies’, but all of these expressions are generally applied towards the same end, that of defining the set of attributes that individuals should possess, or functions that they must be able to do, within their sphere of operation.

A clear and simple definition of competencies has been offered by Bartram et al. as ‘sets of behaviors that are instrumental in the delivery of results’ [2]. In this Universal Competency Framework they make a distinction between competencies (as sets of desirable behaviors); competency potential (individual attributes necessary for someone to produce the desired behaviors); competency requirements (demands made upon individuals in a work setting to behave in certain ways and not in others); and results (actual or intended outcomes of behavior, which have been defined either explicitly or implicitly by the individual, his or her line manager or the organization [2].

According to Khan and Ramachandran, competency refers to the skill itself, and competence to the ability to perform the skill [3]. Another distinction that has been made is the competence primarily concerns the performance requirements of the job, and competencies concern what the job holder brings to the job and encompasses behavior, attitudes, values, ethics [4]. For realizing the true value and potential of competency – ‘alternative lenses’ will need to emerge that may provide developmental support for all arms of a profession—educators, regulators, employers, practitioners, and so forth [5]. The term ‘global health’ has been defined by Koplan et al. as ‘an area for study, research, and practice that places a priority on improving health and achieving health equity for all people worldwide’. This definition focusses on ‘health improvement and health equity’ [6]. Over the last decade, the research work around global health has grown multifold. Apart from university consortiums and professional associations, academic institutions are also exploring the scope of their global health educational programs to meet the demand for a ‘qualified, skilled and competent’ global health professional. Recent research on global health competencies includes the work undertaken by Consortium of Universities of Global Health [7], Association of Schools of Public Health (ASPH) [8], Association of Pacific Rim Universities [9], UK’s Global Health Curriculum Group (GHCG) [10], Canadian Coalition for Global Health Research [11], Global Health Education Consortium (GHEC) and the Association of Faculties of Medicine (AFMC) of Canada’s Global Health Resource Group (GHRG) [12]. However, this exercise of defining competency frameworks/sets has become a one-sided process oblivious to contexts of global health that is ‘health improvement and health equity’ [13]. Additionally, there are several other research studies which is centered around Global Health competencies for UK health professionals [14], for Postgraduate Public Health Education (based on HICs) [15], and for 21st-Century Health Professionals (undertaken by CUGH a Global Health Competency Subcommittee) [16]. Nine core global health research mentoring competencies were also identified for Individuals and Institutions in LMICs [17].

The process of developing the domains and competency statements for global health has been frequently driven in agreement with programs in high income countries (HICs) [13]. This unfortunately has often failed to adequately include the experiences and views of health professionals working in low-income countries (LICs); lower middle-income countries (LMICs) and upper middle-income countries (UMICs). While many global health programs aim to address health inequalities between HICs and low- and middle-income countries (LMICs) [18], there is a need to set the new global health academic programs within the growing trend towards the ‘internationalization of higher education’ [19].

The prevailing focus of the global health programs, dominant in the Global North, is largely about health needs of LMICs, improvement of health worldwide, reduction of disparities, and protection of societies against global threats that disregard national boundaries. It is essential that governments and academic institutions in LMICs, and the Global South in particular, should be engaging on an equal footing towards the development of a mutual understanding of the scope of global health with a shared education, research, and implementation agenda for global health.

Africa can be seen as different from the rest of the world, even from other developing countries which are also affected by the emergence and reemergence of diseases, given that Africa is still threatened by the world’s most acute public health threats, despite medical advancements. Africa accounts for 67% of the global total of new HIV infections [20], 95% of malaria cases [21] and 36% of all TB deaths [22]. Regionally, Africa continues to have the highest rate of under-five mortality and maternal mortality ratio in the world [23]. Despite of having high burden of diseases, Africa can also help other countries learn about epidemic response from its past experiences such as Ebola, Marburg, Yellow Fever and now COVID-19 [24].

As we know, health is also impacted by several variables such as conditions of employment, housing, income, access to education, health, and other critical life resources – which are either unavailable or insufficient in African countries. The work of African scientists – who are working within African region, is often designed through their own lived experiences and gained local knowledge yet major research funding is awarded to HIC institutions as primary recipients [25,26]. Thus, there is a felt need to re-envision global health competencies with respect to the local health needs and availability of resources. In this study we have attempted to find the answer for three questions related to global health competencies within Africa’s context, that is, *‘where are we?’, ‘what is being done?’*, and *‘what should be done?’*. This study can be used as a framework for other lower and middle-income countries for developing competencies for global health within the context of their needs and resources. The findings in this study could serve as a stepping-stone towards building a competent and strong global health workforce which will be seen as an equal and included within the global health community for research and practice.

## Materials and Methods

**Figure 1:**
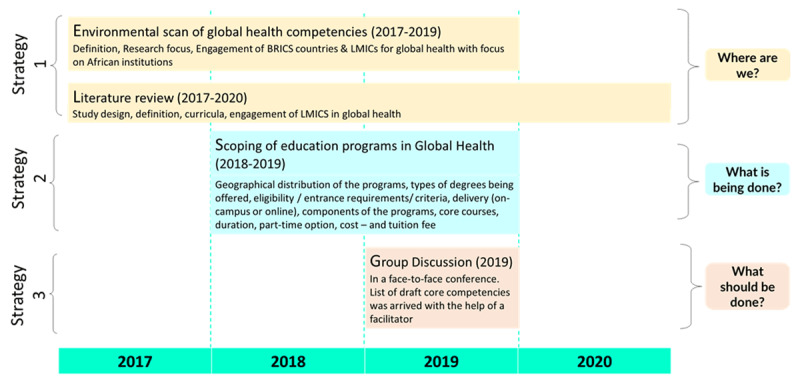
Methodology for developing global health competencies.

This study was undertaken over a period of four years from 2017 to 2020. A three-pronged strategy was undertaken to scan, scope, distil and develop a set of global health domains and competencies for the African region (Figure 1).

As part of the first strategy, an environmental scan of global health competencies was undertaken, where the definition, research focus, engagement of BRICS countries (Brazil, Russia, India, China, and South Africa) and LMICs, and the focus of African institutions for global health were described. A literature review was undertaken from 2017 to 2020 to locate the research focusing on global health in terms of study design, definition, curricula, engagement of LMICs in global health and the focus of African institutions on global health. This activity included development of a search strategy for electronic databases, identifying key journals for hand searching, internet searches of key sites and reference checking. Key words such as ‘global health research definitions’, ‘educational curricula’, ‘engagement of BRICS and LMICs’ were searched in electronic bibliographic databases and registers, hand searches, reference checking and internet searches. This activity helped in conceptualizing ‘*where are we?’* – referring to Africa in the context of global health.

As a second strategy, a scoping review of educational programs in global health was undertaken as a desk review. This exercise was a systematic mapping of Master’s in global health programs offered worldwide during 2018–19. In this exercise, geographical distribution of the programs, types of degrees being offered, eligibility/entrance requirements/criteria, delivery (in-person or online), components of the programs, core courses, duration, format (fulltime or part-time), cost and tuition fee were reviewed. Information was also collected regarding career paths of Master’s in Global Health graduates by searching career information as mentioned on websites of universities in Canada, United States of America, Rwanda, and Switzerland. Brief information regarding undergraduate programs in global health was also collected. Four websites were identified that claimed to present the ‘top’ or ‘best’ programs such as www.mphonline.org, www.best-masters.com, www.healthcarestudies.com, and www.postgrad.com. Global health programs from these websites were used to begin to populate an initial spreadsheet of programs. The spreadsheet was further populated after searching for global health programs at universities known to be involved in global health using Google search engine. Once the global health programs were identified details about each program were collected from the university websites. This activity helped in understanding ‘*what is being done’* – with respect to Africa in the context of global health.

For the third strategy, competency statements in Africa’s context for global health were developed as part of an interest group discussion. The interest group discussion was undertaken with the participants (who registered for the session on global health competencies) at a public health conference held in South Africa in 2019. The list of core competencies was drafted during the interest group discussion which was facilitated by one of the authors. The discussions in the interest group were aided by providing the group with frameworks and reference materials for drafting competencies [7,27]. Bloom’s taxonomy of educational objectives framework was also used to draft global health competencies [29]. The groups also had access to the results of the environmental scan of global health competencies (as mentioned in first strategy). The interest group predominantly comprised of academics and public health professionals within Africa. The profiles of these participants are included in Supplementary Table 1.

*Finalizing competency statements*: Discussion and consensus building exercise was subsequently undertaken wherein the competency statements were revised to avoid duplication or overlap of core competency statements until consensus was reached by the authors. This activity also helped in planning ‘what should be done’ – with respect to global health programs and their competencies in Africa from researchers’ and professionals’ perspective. The summary of methods undertaken for this study over four years have been listed in Table 1.

**Table 1:** Summary of methodology.


METHODOLOGY	SOURCE OF INFORMATION	NUMBERS

Literature review	Electronic bibliographic databases and registers	9 970

	Hand searches	82

	Reference checking	315

	Internet searches	19

Scoping exercise	Global health programs related websites reviewed	4

	Institute/university websites reviewed	73

Interest Group Discussion	Participants	31


Ethical approval for this study was obtained from the Stellenbosch University Health Research Ethics Committee (HREC Reference No: X20/05/022). Verbal consent was obtained from the participants of the conference workshop.

## Results

The results developed from these three strategies have been listed in this section.

*Literature review of global health competencies*: Out of 10 386 citations, 116 global health research studies were included in the review (Supplementary Table 2). The review found that majority of the studies were conducted in the United States of America (USA); 62 for USA most studies focused on education; most research methodology was exploratory and curriculum-based; Africa as a continent and United Kingdom as a country have the same number of global health research studies conducted; 16 each LMICs and BRICS have the lowest classification with regards to global health research.

The term ‘education program in global health’ is broad as it varies from medicine (this includes but not limited to global health residency tracks in various medical specialties, undergraduate and postgraduate programs), nursing, public health, physician assistant, anthropology, and mental health residency programs in various institutions. Although most of the studies included in this review focused predominantly on education, not all detailed their educational curriculum. Some concentrated on the partnership between medical faculty and students, while others were focused on ethics and rotation of global health track students [30,31,32,33,34]. The various curricula were either didactic or experiential learning, which involved peer education or mentoring in domestic and international settings. They were primarily targeted at medical students, clinical specialties, doctors, and residents. The core competencies of global health curriculum specificity vary and still require the necessary harmonization for capacity building for poverty reduction and improved well-being.

*Scoping of educational programs in global health*: The scoping exercise of Master’s level global health programs identified 73 programs on five continents: North America [33]; Europe [23]; Asia [9]; Australasia [4]; and Africa [3]. Whereas one program was offered in partnership between a university in China and the USA; and one in online mode (which can be best described to be multi-country/international/global) [1]. Seventy-seven (77%) percent of the programs identified are offered by universities in Europe and North America, with 29 (40%) offered by universities in the USA. This scoping does not include all global health Master’s programs.

Some universities have more than one type or stream of global health Master’s program. A wide range of degrees are offered in global health including an MPhil in Global Health, although the most common ones are Master of Public Health (MPH) and Master of Science (MSc) programs. A few of the programs identified in the review focus on global health diplomacy or global health leadership. The specific terminology varied to a considerable degree, including ‘MPH in Global Health’ and ‘MPH with a concentration in Global Health.’ Most programs appeared to be professional degrees (e.g., MPH), although a number of research focused programs (e.g., MSc) were identified, however not all MSc in Global Health programs were found to be research focused.

**Figure 2:**
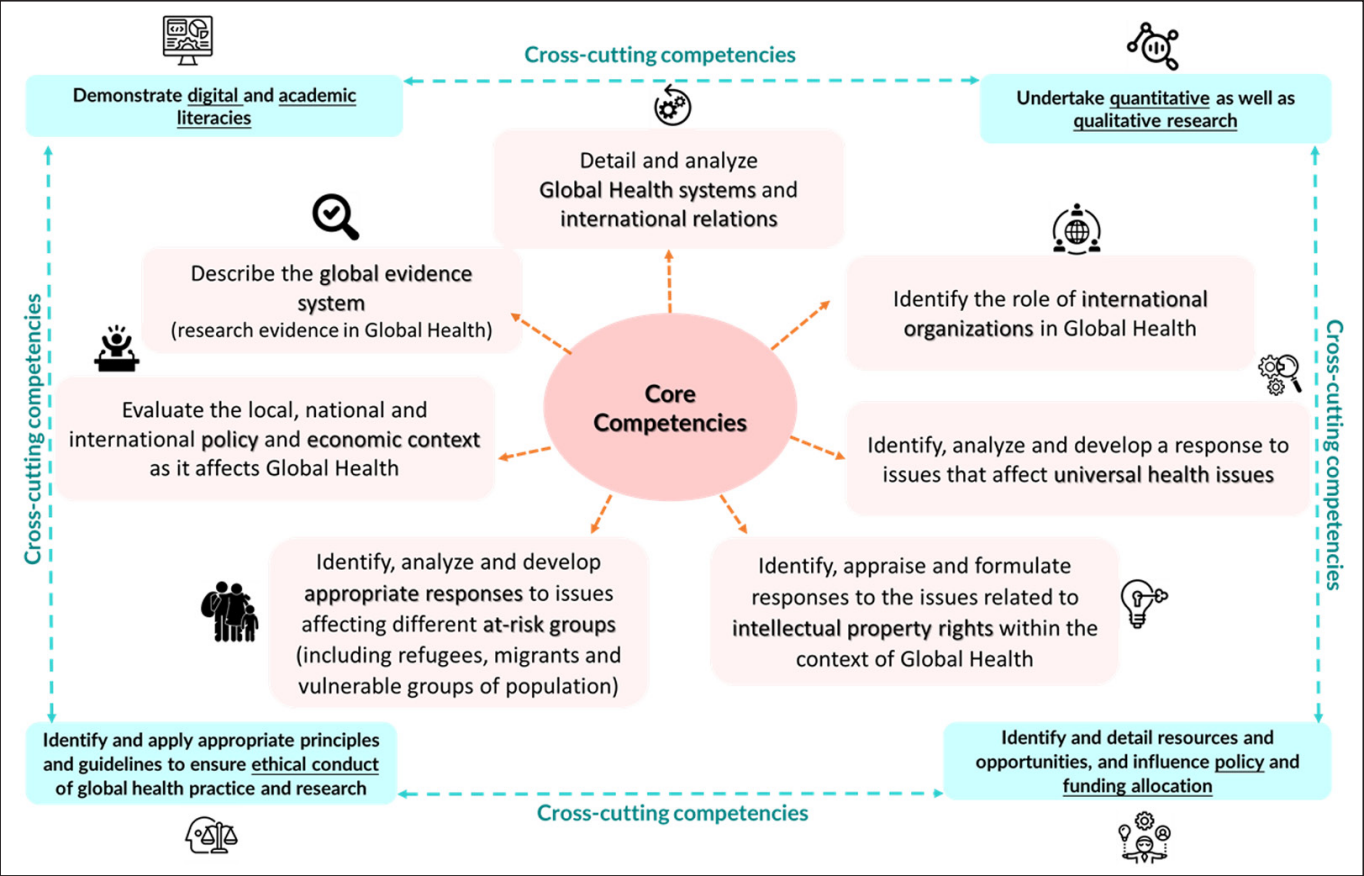
Competency statements for global health within the Africa context.

*Set of competency statements developed in interest group discussion exercise*: The list of competency statements for global health within the African context that were agreed upon by the interest group is provided in Figure 2.

## Discussion

Global health is a field of scholarship and practice seeking to advance health equity by employing a transdisciplinary, inter-sectoral and collaborative approach to address complex health and social problems that cross-national borders and are impacted by transnational forces [35]. In practice, the Global North has been dominant in directing and setting competency guidelines for the global health community [36]. However, times are changing and equal representation of the voice of the Global South and LMICs should be ensured and evidenced in major global health associations increasingly developing these competencies and guidelines.

In 2012, global health competencies for nurses in the Americas were developed to guide faculty deliberations about global health competencies that should be incorporated in the nursing curricula [37]. Similarly, the global health community in Africa needs to develop and adopt a global health competency framework tailored to the needs of Africa. In 2014, the University of Global Health Equity was established in Kigali, Rwanda – to advance global health delivery, and to train a new generation of global health leaders who are equipped in not just building, but sustaining effective and equitable health systems [38]. Such initiatives are needed in greater numbers in African region.

Currently, there are no imperatives or incentives for departments/universities that offer global health programs in the African region/LMICs to collaborate or share resources. Most of the public health/community health/global health departments effectively function in isolation in Africa. A culture of collaboration among these departments would encourage a sharing of best practices in tuition, scholarships, curriculum development, widening the faculty resource pool and placement & internship opportunities for global health graduates. Currently, global health graduates are employed in public, private and nongovernmental sectors, in teaching, research, consulting and implementation roles. However, there are no well-defined career pathways for global health graduates, which is a significant barrier for interested candidates who wish to undertake these courses and work in the global health sector.

We reviewed the match of the global health competency statements developed within the African context with the recently published research articles by Jacobsen et al. for undergraduate minors in global health [39], and postgraduate Master of Science (MSc) and Master of Arts (MA) Degrees in Global Health [40] (Table 2) – as this research work focusses on undergraduate and postgraduate programs in Africa. For undergraduate minors three competencies viz., detail and analyse global health systems and international relations; evaluate the local, national and international policy and economic context as it affects global health; identify and detail resources and opportunities, and influence policy and funding allocation – were observed to be matching. For Master’s degrees in global health four competency statements viz., detail and analyse global health systems and international relations; evaluate the local, national and international policy and economic context as it affects global health; identify, analyse and develop a response to issues that affect universal health issues; identify, analyse and develop appropriate responses to issues affecting different at-risk groups (including refugees, migrants and vulnerable groups of population) – were found to be matching.

**Table 2:** Match of the global health competency statements developed within African context with recently published research studies in 2020.


COMPETENCY STATEMENTS FOR GLOBAL HEALTH WITHIN THE AFRICAN CONTEXT	JACOBSEN ET AL. (2020) – UNDERGRADUATE MINORS IN GLOBAL HEALTH	JACOBSEN ET AL. (2020) – MS AND MA DEGREES IN GLOBAL HEALTH

**Core Competencies:**		

1. Detail and analyse global health systems and international relations	**x**	**x**

2. Describe the global evidence ecosystem (research evidence in global health)	**–**	**–**

3. Identify the role of international organizations in global health	**–**	**–**

4. Evaluate the local, national and international policy and economic context as it affects global health	**x**	**x**

5. Identify, analyse and develop a response to issues that affect universal health issues	**–**	**x**

6. Identify, analyse and develop appropriate responses to issues affecting different at-risk groups (including refugees, migrants and vulnerable groups of population)	**–**	**x**

7. Identify, appraise and formulate responses to the issues related to intellectual property rights (TRIPS etc.) within the context of global health	**–**	**–**

**Cross-cutting/intersectoral competencies:**		

1. Demonstrate digital and academic literacies	**–**	**–**

2. Undertake quantitative as well as qualitative research	**–**	**–**

3. Identify and detail resources and opportunities, and influence policy and funding allocation.	**x**	**–**

4. Identify and apply appropriate principles and guidelines to ensure ethical conduct of global health practice and research	**–**	**–**


Key: **x:** covered, **–:** not covered. Source: Author’s own.

Our work has a few limitations. In this review we mainly focused on global health competencies within the African context and have not detailed sub-competencies, nor undertaken a more detailed analysis of the depth of the identified competency domains. We plan to conduct the detailed analysis in the context of depth of global health competency domains for both undergraduate and postgraduate students. For the interest group discussion most participants came from universities based in South Africa with some participants from other African countries. However, this was not an absolute representative sample for whole of African region.

Within Africa there are five distinct regions, that is, West, East, North, Southern and Central. Countries that deem themselves developed also need to learn from others, as seen during the COVID-19 pandemic [41]. Countries of the Global South need capacity strengthening while those in the North are assumed to have a strong capacity regardless of any health issues [25].

## Conclusion

The majority (92.7%) of the global/international health programs are offered in North America, Europe and the Western Pacific region [42]. Traditionally, global health expertise and thus research flows from the Global North to Global South and this is reflected in research, training and technical assistance. Thus, there is a need for developing a more explicit lens around global health competencies for the African region. Education needs for global health students are different for African institutions as compared to other developed regions of the world. We hope that the concept of this research study can be used for developing global health competencies in other regions/countries of the world.

There is a need to enable Higher Education Institutions (HEIs) from the Global South to offer global health qualifications with a set of competencies that better approximate solutions to contextualised problems– not only for students from the Global South but also from Global North. The global health competencies developed in this research study will enable African HEIs to offer global health education in a more pragmatic manner.

## Supplementary Table

**Supplementary Table 1:** Profiles of participants in interest group exercise undertaken in public health conference organized by PHASA in South Africa in 2019.


SR. NO.	AFFILIATION	AGE GROUP	COUNTRY OF ORIGIN

1	Stellenbosch University	46–65	South Africa

2	Stellenbosch University	>25	South Africa

3	Stellenbosch University	46–65	South Africa

4	Stellenbosch University	26–35	Zimbabwe

5	Stellenbosch University	>25	Malawi

6	Stellenbosch University	26–35	Nigeria

7	Teaching Nations	46–65	South Africa

8	Teaching Nations	46–65	South Africa

9	Stellenbosch University	26–35	South Africa

10	Stellenbosch University	36–45	Nigeria

11	Stellenbosch University	46–65	South Africa

12	Stellenbosch University	36–45	South Africa

13	Stellenbosch University	46–65	South Africa

14	Stellenbosch University	>25	South Africa

15	Stellenbosch University	36–45	South Africa

16	Stellenbosch University	26–35	South Africa

17	Health Systems Trust	46–65	South Africa

18	Health Systems Trust	46–65	South Africa

19	University of Western Cape	46–65	South Africa

20	Stellenbosch University	>25	South Africa

21	Stellenbosch University	>25	Belgium

22	University of Cape Town	26–35	South Africa

23	Stellenbosch University	26–35	South Africa

24	University of Cape Town	26–35	South Africa

25	Stellenbosch University	46–65	South Africa

26	University of Cape Town	26–35	South Africa

27	University of Witwatersrand	26–35	South Africa

28	USAID TB Project	26–35	South Africa

29	University of Witwatersrand	36–45	Ghana

30	National Department of Health, South Africa	46–65	South Africa

31	Raleigh Fitkin Memorial Hospital	36–45	Swaziland

32	University of KwaZulu Natal	36–45	South Africa


**Supplementary Table 2:** Search strategy.


NUMBER	SEARCH CRITERIA

10 386	citations

8 274	on account of duplicates

3 134	screening the titles, remained

1 452	abstracts were obtained

542 (59.5%)	met inclusion criteria

910	Full reports were obtained of 910

184	726 excluded (editorials, commentaries, conferences…)

35	studies not retrieved – unavailable at libraries

28	excluded with reasons

**116**	**classified Type A studies & reported on**

